# Effectiveness of Behavior Change interventions for smoking cessation among expectant and new fathers

**DOI:** 10.1093/eurpub/ckac129.038

**Published:** 2022-10-25

**Authors:** S Khanal Bhattarai, C Miani, E Finne, JM Morawe, M Boeckmann

**Affiliations:** 1 School of Public Health, University of Bielefeld, Bielefeld, Germany; 2 Faculty of Human and Health Sciences, University of Bremen, Bremen, Germany

## Abstract

**Background:**

The perinatal period is an optimal time to intervene for achieving smoking cessation in expectant parents and offers multiple health benefits for women and the newborn. While Behavior Change Technique (BCT) interventions are a promising approach to support pregnant smokers to quit smoking, effectiveness of these interventions among expectant and new fathers is not equally well documented. Better understanding of the potential utility of these BCT interventions for this group is important for the development of effective gender-sensitive programmes.

**Methods:**

This systematic review examines the existing evidence on effectiveness of BCTs on smoking cessation outcomes when offered to expectant and new fathers (child < 1 year) both through individual and/or couple-based interventions. Eight databases were searched for peer-reviewed articles. Studies were subjected to systematic retrieval and quality-assessment by two independent reviewers.

**Results:**

We identified 9 randomised control trial studies (including 4,681 men) that fulfilled the inclusion criteria. In terms of quit outcome data, 8 studies reported biochemically verified quit rates for men. While 5 BCT interventions targeted expectant/new fathers, 3 were directed to couples and 1 primarily focused on women with a component directed at men. Though most of the interventions were found to be effective, they showed small significant positive effects on cessation outcomes. Findings are suggestive of gender specific interventions being more likely to have positive outcomes. High heterogeneity across the studies made it difficult to determine the most effective BCT approach.

**Conclusions:**

This review suggests that use of BCT interventions for smoking cessation among expectant and new fathers is effective in achieving positive quit rates; however, these studies are limited. Further research is needed to determine the most effective BCT approach associated with smoking cessation among this group.

**Key messages:**

• BCT interventions for smoking cessation among expectant and new fathers are a promising approach to increase quit rates.

• Future research needs to develop evidence based BCT interventions for smoking cessation specifically targeting expectant and new fathers to inform policy and practice.

